# Pandemic Pedagogy: Perception of Nursing students’: A cross-sectional study

**DOI:** 10.12688/f1000research.109789.2

**Published:** 2022-07-27

**Authors:** Prima Jenevive Jyothi D'Souza, Anil Raj Assariparambil, G Muthamilselvi, Veena M Joseph, Linu Sara George

**Affiliations:** 1 Assistant Professor, Department of Fundamentals of Nursing, Manipal College of Nursing Manipal, Manipal Academy of Higher Education, Udupi district, Karnataka, 576104, India; 2 Assistant Professor, Department of Medical Surgical Nursing, Manipal College of Nursing Manipal, Manipal Academy of Higher Education, Udupi District, Karnataka, 576104, India; 3 Professor and principal, Vinayaka Mission’s College of Nursing, Vinayaka Mission’s Research Foundations,, Puducherry, Tamil Nadu, India; 4 Assistant Professor, College of Nursing, Gulf Medical University, Ajman, United Arab Emirates; 5 Professor & Head, Department of Fundamentals of Nursing, Manipal College of Nursing Manipal, Manipal Academy of Higher Education, Udupi district, Karnataka, 576104, India

**Keywords:** Nursing, Perception, Pandemics, Students, Teaching

## Abstract

Coronavirus disease 2019 pandemic impacted across the globe disrupting all sectors including the higher education universities. Nursing institutions faced various challenges due to the pandemic restrictions, of which the abrupt shift of implementing the curriculum to online mode posed a major challenge to both the teachers and the students. To assess nursing students’ perception of pandemic pedagogy and the challenges faced in online teaching-learning, this cross-sectional survey was conducted among 982 undergraduate nursing students from three Deemed to be University nursing institutions of Southern India. Institutional Ethics Committee approval (IEC 444/2020), permission from the heads of the institutions and study participant’s consent was obtained. Data was collected using an online survey questionnaire which had three domains, including student-related (19 items), teacher-related(5 items), and physical learning environment-related factors (11 items). The reliability was established using Cronbach’s Alpha (0.86). Explored the favouring, hindering factors and challenges faced during the emergency remote teaching with open-ended items. The overall mean score of perceptions on pandemic pedagogy was 89.03±10.03. Sixty-three percent of students had a total perception score above 87 which indicates that they preferred online learning during the pandemic whereas 45% preferred classroom learning. There was a significant difference in the total perception scores and the years of study( F (3, 978) = 4.96, p = 0.002). The factors favouring online learning were, an opportunity to view the recorded classes even after the live classes’ (n=165), and ‘more time to spend for learning activities’ (n=152). Factors that hindered the learning or the challenges faced were poor network connectivity (n=451), and lack of opportunity for group study (n=326). Students favoured online learning during the pandemic; however, there were several challenges. The educational institutions need to prepare themselves to overcome this and focus on a blended learning curriculum.

## What is already known about the topic?

• COVID-19 pandemic has disrupted educational activities worldwide.
• Higher education institutions, including nursing, have quickly moved to remote teaching-learning for uninterrupted education.
• There is a gap in the knowledge of emergency remote teaching for the nursing curriculum during the COVID-19 pandemic.

## What this paper adds?

• This study describes how nursing students perceived remote learning and the challenges faced during the COVID-19 pandemic.
• The findings of the study contribute to nursing education, emphasizing the need of remote teaching-learning for uninterrupted learning and prevent the spread of infection by staying safe at home.
• The incorporation of blended learning post-pandemic helps nurse educators and the policymakers to take appropriate steps in escalating the nursing education in the country to greater heights.

## Introduction

The outbreak of Coronavirus Disease 2019 (COVID-19) hampered educational activities worldwide. Educational institutions in most countries had to go remote instructions from face-to-face teaching during lockdown and after. When COVID-19 crisis would end in uncertain and colleges started functioning as before has led to continuing e-learning in many parts of the world indefinitely until the pandemic comes under control.

What we experienced during the COVID-19 was unprecedented. Nursing institutions faced a unique challenge as they had a significant role in bringing up the next generation of care providers ( Dewart *et al.*, 2020). COVID-19 pandemic had pushed us towards e-learning from traditional classroom learning ( Hodges *et al.*, 2020). The abrupt interruption of the well-planned academic activities in the face-to-face classes and the shift to remote teaching and learning was challenging for both teachers and students due to the technology and its accessibility.

Nursing education focuses on the development of knowledge and clinical competency for providing quality care to the patients. During the course of study, theory and practicum go hand-in-hand for students and thereby bridge the gap between theories and practice. Students acquire their clinical skills by practicing in both laboratory and hospital. Getting adequate clinical experiences would improves the skills in performing nursing procedure, rather than enhancing critical thinking and clinical decision-making abilities. As the government declared the closure of educational institutions due to the pandemic, students had to move to their homes without knowing about their return to the colleges. Both students and teachers assumed that they would return at the earliest, but the pandemic kept them in their homes for more than six months.

All the nursing schools in India quickly moved on to emergency remote teaching from face-to-face classes. The institutions adopted synchronous and asynchronous technology based on convenience and availability. The synchronous technology included live interactions between teacher and students during online classes, whereas the asynchronous had a time gap between the instructor and its recipient ( Finkelstein, 2006). The implementation of emergency remote teaching helped to continue learning outside educational institutions, keeping students and teachers safe in their homes. During this phase, students might have had varied experiences and faced various challenges due to the changes in learning environment. Before the pandemic, most of the teaching-learning activities occurred either in formal classrooms or laboratories under teacher’s constant mentoring, whereas in the mode of emergency remote teaching, the students had to take the responsibility for learning themselves.

A study conducted by Dvořáková *et al.* (2021) on university students’ perception of remote learning during the first wave of the COVID pandemic. A total of 68 under-graduates and 31 post-graduates were included in the online survey using MS forms. Among 99 participants, the average overall score for satisfaction with emergency remote teaching was 4.07 out of 5. Eighty percent of students revealed that during remote learning students missed the opportunities for classroom discussions and interaction with classmates and teachers. Regarding their stress level during remote learning, 48% expressed that they were partly stressed, and 19% expressed being more stressed than regular face-to-face teaching.

The emergency remote teaching or pandemic pedagogy in nursing and other professions has not been much explored. The objective of the study is to assess the perceptions of the undergraduate students of nursing on the pandemic pedagogy and identify the challenges they faced in remote learning.

## Methods

### Design and sample

This cross-sectional survey was conducted among undergraduate nursing students studying in the deemed to be university nursing institutions, under the University Grants Commission of India, Southern India.

### Setting and data collection

Investigators obtained the approval from the Institutional Ethics Committee (IEC 444/2020) for conducting the study and permission from the heads of the institutions. The study was registered under the clinical trial registry of India (CTRI/2020/08/027047).

All the deemed to be universities nursing institutions of Southern India were approached and included only those permitted to conduct the study. The students undergoing a four-year undergraduate nursing degree programme were included in the study. The students were sent home from the institutions under lockdown, and all three nursing institutions moved on to emergency remote teaching. Since the institutions did not have remote teaching before the pandemic, it was a sudden change for both teachers and students. Initially, teachers themselves explored various platforms and later they were oriented to remote teaching platforms like Microsoft teams, Impartus and Edmodo by the institutions too. The nursing institutions offered around three to five hours of remote instruction during the pandemic.

An online survey with written consent form was sent to all the participants via Google form to their contact details submitted to the institution and the students consented to participate in the study after reading the participant information sheet provided in the form.

### Sample size and sampling technique

**Figure 1.**
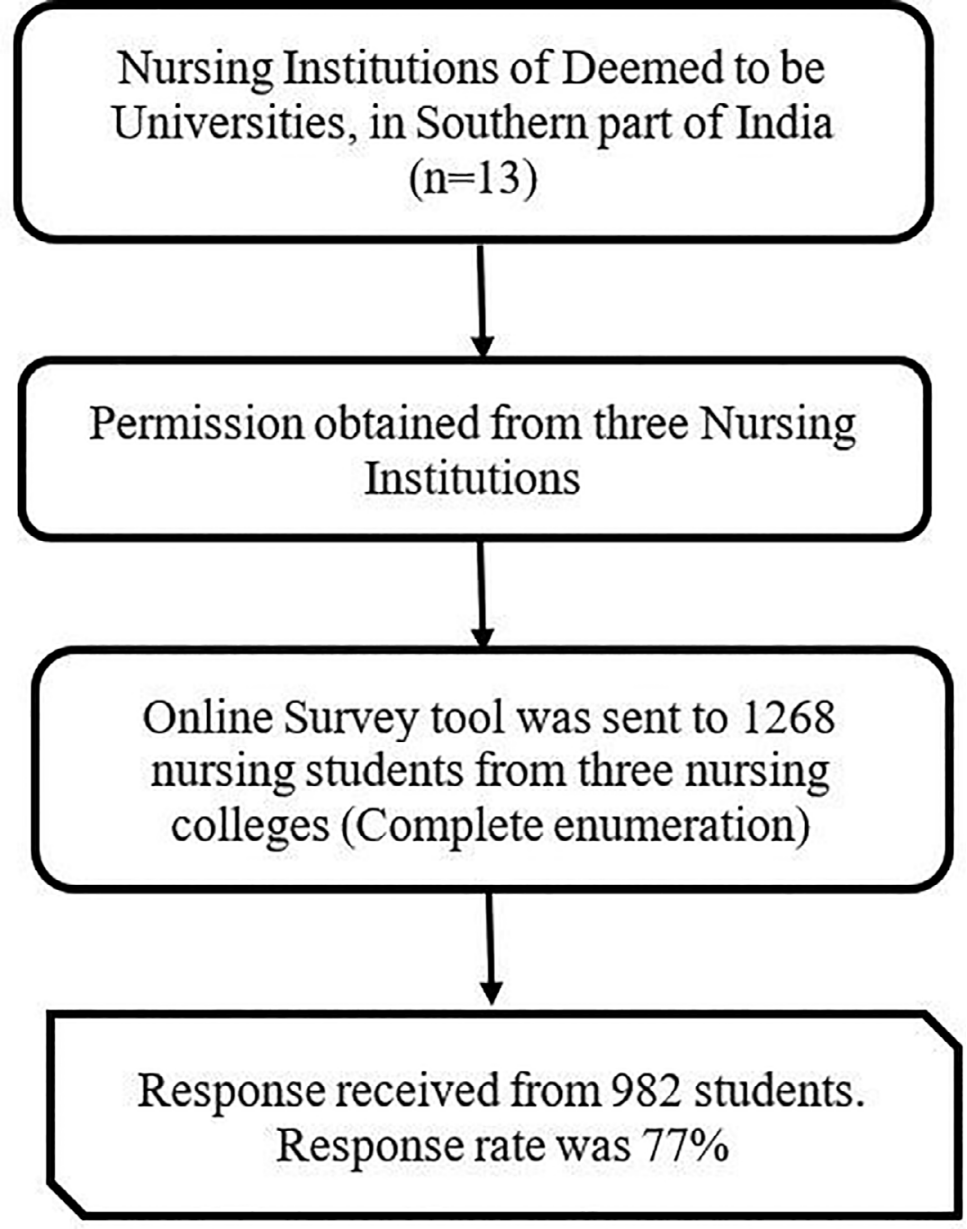
A schematic flow chart representing sample selection.

A complete enumeration of all the nursing institutions of the deemed to be universities of Southern India was the sampling technique considered in the study. However, out of 13 nursing institutions of the Deemed Universities in the selected geographical area, three nursing colleges accepted to participate in the study. The online survey questionnaire was sent to all the students of the three colleges and requested them to participate in the study if they were willing to. Figure 1 depicts the schematic flow chart representation of the sample selection.

### Variables and measures

#### Demographic

A demographic questionnaire was used for collecting the information on age, year of study, area of residence, mode of access to e-learning, and the device used.

#### Perception of pandemic pedagogy

The perception of students on the pandemic pedagogy was assessed using a four-point Likert scale. It consisted of 35 items with four open-ended questions. The questionnaire had three domains, including student-related (19 items), teacher-related (5 items), and physical learning environment-related factors (11 items). The response options for each item were: Strongly Agree (SA), Agree (A), Disagree (D), and Strongly Disagree (SD) with scores of 4, 3, 2, 1, respectively. The total score ranged from 35 to140, with a cut-off of > 87 as an indicator of favouring remote learning. The scores less than 86 indicated that the students favoured the face-to-face classroom learning during the pandemic. The open-ended questions explored the favouring factors and the challenges students faced during the emergency remote teaching.

The investigators developed this survey questionnaire after reviewing the available literature and taking opinions from the students. It was validated by experts from the field of nursing and medical education. The reliability was established by administering the scale to 20 Post Basic BSc. nursing students and the Cronbach’s Alpha computed was 0.86.

### Statistical analysis

The data analysis of the study was carried out using SPSS version 16 (SPSS, RRID:SCR_002865). The demographic variables and the perceptions of the students on remote learning were expressed in frequency and percentage. The mean and the standard deviation of the perception score based on the year of study is described. To find the difference in the perception score between the years of study, ANOVA was used and post hoc comparisons was performed using the Hochberg’s GT2 test. Independent sample t-test was performed to find the difference between the areas of residence and the perception score. The factors facilitating the pandemic pedagogy and the challenges faced were described in frequency.

## Results

### Sample characteristics

The characteristics of the nursing students who participated in the pandemic pedagogy perception survey are summarized in Table 1. The mean age of student nurses was 19.8 years ( *SD*=1.4). The common platforms used by the institutions for the remote pedagogy were Zoom (34.3%), MS Teams (34.3%), Webex (8.2%), Google meet (7.2%), Edmodo (7.1%), Impartus (5.8%), and Google classroom (2.7%).

**Table 1.** A description of sample characteristics (N=982).

Sample characteristics	Frequency (f)	Percentage (%)
*Year of study*		
First	127	12.9
Second	236	24.0
Third	248	25.3
Fourth	***371***	***37.8***
*Area of residence*		
Rural	***530***	***54.0***
Urban	432	46.0
*Type of device used for remote learning*		
Mobile phone	***866***	***88.2***
Computer	20	2.0
Tablet	6	0.6
Both Mobile phone and Computer	90	9.2
*Network coverage at the place of stay*		
Good	206	21.0
Average	***651***	***66.3***
Poor	125	12.7
*The orientation is given by the institution on the use of the remote teaching platform*		
Yes	***888***	***90.4***
No	94	9.6

### Nursing students’ perception of pandemic pedagogy

The overall mean score of the perceptions of students on the pandemic pedagogy was 89.03±10.03. The year-wise mean scores are given in Table 3. 63% of students had a total perception score above 87 indicates that they preferred remote learning during the pandemic, whereas 45% preferred face-to-face learning in the classroom. The response for the individual items on the perception scale is summarized in Table 2.

**Table 2.** Description of nursing students’ perception of pandemic pedagogy (N=982).

Sl. No.	Item	Perception			
		Strongly agree	Agree	Disagree	Strongly disagree
		f (%)	f (%)	f (%)	f (%)
	***Students related factors***				
1.	Remote classes helped me to accomplish the learning objectives.	84 (8.6)	715(72.8)	129 (13.1)	54 (5.5)
2.	I was able to concentrate better during the remote classes compared to the face-to-face classes.	53 (5.4)	354 (36.0)	425 (43.3)	150 (15.3)
3.	The new teaching environment improved my self-directed learning skills.	71 (7.2)	638 (65.0)	227 (23.1)	46 (4.7)
4.	The remote classes were more interactive than the classroom learning.	48 (4.9)	320 (32.6)	465 (47.4)	149 (15.2)
5.	The remote class timings were convenient than the classroom learning.	2 (0.2)	702 (71.5)	213 (21.7)	65 (6.6)
6.	I was able to communicate with my classmates well during this pandemic time like that in the classroom.	85 (8.7)	428 (43.6)	362 (36.9)	107 (10.9)
7.	I never got a sense of isolation by attending the remote classes being away from the regular classroom.	83 (8.5)	564 (57.4)	262 (26.7)	73 (7.4)
8.	Being away from the regular classroom, I needed to have more accountability to achieve the learning outcomes.	103 (10.5)	729 (74.2)	126 (12.8)	24 (2.4)
9.	Being away from the regular classroom, I needed to take more responsibility to achieve the learning outcomes.	158 (16.1)	713 (72.6)	86 (8.8)	25 (2.5)
10.	The remote learning helped me to understand the course concepts as good as the regular classroom.	74 (7.5)	501 (51.0)	319 (32.5)	88 (9.0)
11.	Virtual demonstration of nursing procedures helped me to develop my clinical skills as in the regular teaching.	67 (6.8)	484 (49.3)	328 (33.4)	103 (10.5)
12.	Being away from the clinical setting I felt I am not confident of performing the procedures that I missed to practice.	202 (20.6)	613 (62.4)	123 (12.5)	44 (4.5)
13.	My clinical skills have been affected by being away from the clinical setting for a longer time.	259 (26.4)	577 (58.8)	114 (11.6)	32 (3.3)
14.	Being away from the clinical setting, I could not incorporate the theory into practice and it hindered my learning.	154 (15.7)	665 (67.7)	133 (13.5)	30 (3.1)
15.	I could not develop the clinical decision-making skills being away from the clinical setting.	154 (15.7)	674 (68.6)	129 (13.1)	25 (2.5)
16.	Being away from the institution for a long duration and attending classes remotely has made me stressed.	187 (19.0)	519 (52.9)	222 (22.6)	54 (5.5)
17.	Engaged in the remote learning, I am worried about the depth of my knowledge of the course content.	149 (15.2)	634 (64.6)	169 (17.2)	30 (3.1)
18.	Being away from the regular classes has affected my overall development.	161 (16.4)	566 (57.6)	212 (21.6)	43 (4.4)
19.	The regular classroom learning gave opportunities for the extracurricular activities.	214 (21.8)	619 (63.0)	107 (10.9)	42 (4.3)
	***Teacher related factors***				
20.	The remote classes gave me enough opportunity to clarify doubts with the teacher.	121 (12.3)	604 (61.5)	205 (20.9)	52 (5.3)
21.	Teachers motivated me during the remote classes though I was away from the institution.	176 (17.9)	694 (70.7)	85 (8.7)	27 (2.7)
22.	During the remote classes, the teacher was able to give attention to the individual students as in the physical classroom.	80 (8.1)	552 (56.2)	272 (27.7)	78 (7.9)
23.	The instructions given during the remote classes were clear as in the physical classroom.	85 (8.7)	608 (61.9)	232 (23.6)	57 (5.8)
24.	The teacher kept the learners active throughout the class.	101 (10.3)	701 (71.4)	138 (14.1)	42 (4.3)
	***Physical learning environment-related factors***				
25.	The remote learning was safer amid the COVID pandemic.	288 (29.3)	608 (61.9)	56 (5.7)	30 (3.1)
26.	The remote classes kept me engaged so much that I did not have time to think unnecessarily about the COVID-19.	153 (15.6)	627 (63.8)	157 (16.0)	45 (4.6)
27.	The mode of remote teaching gave adequate opportunities for the instructor feedback as in the physical classes.	88 (9.0)	650 (66.2)	194 (19.8)	50 (5.1)
28.	The home atmosphere was not suitable for the teaching and learning process.	164 (16.7)	475 (48.4)	243 (24.7)	100 (10.2)
29.	The software used for the remote learning was user friendly.	134 (13.6)	690 (70.3)	115 (11.7)	43 (4.4)
30.	The remote learning allowed sharing the teaching-learning material on the same platform.	139 (14.2)	711 (72.4)	100 (10.2)	32 (3.3)
31.	Network/internet issues interfered with my remote learning.	339 (34.5)	508 (51.7)	96 (9.8)	49 (4.0)
32.	Unfamiliarity with the use of technology made the remote learning difficult.	107 (10.9)	519 (52.9)	274 (27.9)	82 (8.4)
33.	The use of the learner engagement applications (Kahoot, Mentimeter, etc.) kept me active during the remote class.	125 (12.7)	581 (59.2)	233 (22.7)	43 (4.4)
34.	The technical skills of the teachers helped to have an uninterrupted classes.	108 (11.0)	678 (69.0)	158 (16.1)	38 (3.9)
35.	The remote classes were interesting, and I enjoyed learning.	102 (10.4)	524 (53.4)	261 (26.6)	95 (9.7)

### A comparison of nursing students’ perception of pandemic pedagogy with years of study

The three domains included in the perception tool were student-related, teacher-related, and physical learning environment-related factors. This section of the article discusses the comparison of domains wise analysis with the students as per their years of study. The comparison table depicted in Table 3 reveals no changes between the mean values of the domain scores across the different years of study. To make the comparison of nursing students’ perception on pandemic pedagogy with years of study, ANOVA was computed and it revealed that there was a significant difference ( *M*=89.03, *SD*=10.03, F (3, 978)=4.96, p=0.002) in the total perception scores and the years of study. The post hoc comparisons using the Hochberg’s GT2 test indicated that the mean perception score of the first-year students ( *M*=86.48, *SD*=10.95) was significantly different than the second-year students ( *M*=90.97, *M*=9.83). However, the third ( *M*=89.43, *SD*=9.11) and the fourth year ( *M*=88.65, *SD*=10.26) students’ perception scores did not significantly differ from the first and the second-year students.

**Table 3.** A comparison of nursing students’ perception of pandemic pedagogy mean score with the years of study (N=982).

Year of study	Student-related factors	Teacher-related factors	Physical learning environment-related factors	Overall	Mean Square	df	f	p
	(Min 19-Max 76)	(Min 5-Max 20)	(Min 11-Max 44)	(Min 35-Max 140)				
	Mean±SD	Mean±SD	Mean±SD	Mean±SD				
1 ^st^ Year *(n=127)*	43.89±5.87	13.59±2.56	29.31±4.04	86.80±10.12	493.75	3,978	4.96	0.002 ^*^
2 ^nd^ Year *(n=236)*	46.0±5.26	14.51±2.64	30.24±3.43	90.95±9.27				
3 ^rd^ Year *(n=248)*	45.51±5.87	14.02±2.37	29.37±3.92	88.91±9.80				
4 ^th^ Year ( *n=371)*	45.28±6.25	14.06±2.53	29.30±3.50	88.65±10.39				
Overall ( *N=982)*	45.38±5.91	14.10±2.54	29.54±3.68	89.03±10.03				

^*^ Significant at the level of p<0.05.

### Comparison of nursing students’ perception of pandemic pedagogy with the area of residence

A comparison of the perceptions of the students of nursing on the pandemic pedagogy with the area of residence has been described in Table 4. To make the comparison an independent sample ‘t’ test was used to compute. The findings reveal that there is no significant difference in the perception scores of the students residing in the rural ( *M*=88.68, *SD=*9.86) and the urban ( *M*=89.43, *SD=*10.23) areas; t (1.16), p=0.0243.

**Table 4.** A comparison of perceptions of students of nursing on pandemic pedagogy with the area of residence (N=982).

Area of residence	n	Mean±SD	df	t	p
Rural	530	88.68±9.86	980	1.16	0.243
Urban	452	89.43±10.23			

### Factors favouring pandemic pedagogy

The participants were asked to express their views on the factors that favoured remote learning during the COVID-19 pandemic. The responses are summarized descriptively, and the major aspects revealed were: ‘an opportunity to view the recorded classes even after the live classes’ (n=165), ‘more time to spend for learning activities’ (n=152), ‘ample opportunity to clarify doubts as same as a physical classroom’ (n=139) and ‘able to concentrate well as I felt that the teacher is directly interacting’ (n=110). Other opinions were, “ *flexible and convenient class timings motivated to do self-directed learning, study materials were made available immediately after each lecture helped in completing studies on time, in the current situation of a pandemic being along with parents were relaxing and stress-free and which helped in better learning, training sessions arranged by the college helped a lot in handling the remote teaching-learning platform efficiently.”*

### Challenges faced during pandemic pedagogy

The descriptive summarization of the responses towards the aspect of factors that hindered the learning or the challenges faced in remote teaching-learning during the COVID-19 pandemic is discussed in this section. The majority of students of nursing responded that the internet issues, either due to poor network coverage, bad weather or monsoon, were a major concern as they could not understand the topics discussed (n=451). The participants also quoted a few other challenges: ‘could not have group study and discussion which was practiced in the college campus’ (n=326), ‘Clinical skills cannot be taught well through the remote teaching platforms’(n=315), ‘attending the classes through the mobile phone was difficult as often getting tired (headache, eye issues) due to the constant use’ (n=262), ‘less motivation to learn being away from the college’ (n=205), and ‘not able to concentrate being at home’ (n=135). Some of them also had mentioned the *unavailability of library facilities and fewer opportunities to maintain the interpersonal relationship with the teachers and the friends* as the challenges faced during this pandemic time.

### Opinion on future directives for classes during the post-pandemic period

Participants were asked to express their opinion on having a few hours of remote instructions along with the face-to-face classes during the academic year post pandemic. The descriptive summary of the open-ended questions reveals that the future directions on the remote instructions were inconclusive. Most of them preferred to be at the safe places during the pandemic and continue with the remote learning. Under the favouring factors and the challenges faced during the remote instructions, there is a mixture of opinions about the future direction of remote learning. As the curriculum demands hands-on clinical experience through direct patient care, they opined that it was a major missing. A few of them even suggested that the theory hours in future could be a mixture of both the online and the offline classes, whereas a few felt that there were a lot of negative impacts of remote learning since it had its pros and cons. To list a few physical ill effects, no opportunity for co-curricular activities, less opportunity to be together and interact with the friends and the teachers. Unanimously most of them expressed that though there were pros and cons for the remote instructions, the biggest challenges were the network issues, impact of weather over electricity, and data connectivity.

## Discussion

This paper intended to study undergraduate nursing student’s perception towards the remote learning adopted during the COVID-19 pandemic. The institutions of nursing education quickly moved on to the mode of remote learning to have uninterrupted learning during the lockdown. This was an unprepared and sudden change in the education for which the students and the teachers were not ready. Due to a lack of preparedness, the teachers and the students faced many challenges, especially during the transition period. The present study reveals that 81.4% of the participants (72.8% A and 8.6% SA) agreed that the remote instructions helped to accomplish the learning objectives. The most students (43.3% D, 15.3% SD) disagreed that they could concentrate better during the remote instructions compared to the face-to-face teaching and the remote instructions were more interactive than the classroom learning (47.4% D, 15.2% SD). Though there was an online learner engagement with the remote learning, the majority worried (64.6%) about the depth of knowledge of the course content.

Bali and Liu (2018) explored higher education students’ perception and indicated that social presence, social interaction, and satisfaction were higher in the face-to-face learning than the remote learning. However, a few students preferred the remote learning as the technology led them to be innovative. The student engagement remains a challenge in the remote learning. Engaging the students and enhancing the student-learning may not be as effective in the remote learning as in the classroom learning. The students may not be able to discuss and have their doubts that arise during the remote classes cleared ( Alturise, 2020). In the present study, the students perceived that they were kept active throughout the class (71.4% A, 10.3% SA) with the use of learner engagement applications (59.2% A, 12.7% SA). The remote teaching-only classes may affect the student performance, especially for those who are already academically struggling. The face-to-face instructions would enable the academic performance of students than the online courses ( Xu & Jaggars, 2014). In the present study, there was a mixed response from the participants on the aspect of opportunity to communicate with classmates well during this pandemic time like that happened in the classroom (43.6% A, 36.9% D). The most of them (57.4 % A, 8.5% SA) agreed that they never got a sense of isolation while attending the remote classes being away from the regular classroom and 63% agreed that the opportunities for extracurricular activities were more in the regular classroom learning. The majority of them (71%) perceived that the teachers kept the learners active throughout the class and 59% agreed that the use of learner engagement applications (Kahoot, Mentimeter, etc.) kept them active during the remote class.

The remote learning would hinder in acquiring clinical skills. A study conducted among the dental students and the staff reported that the closure of teaching clinics would affect the students’ clinical competence ( Loch *et al.*, 2020). In the present study, the most students felt that they were not confident to perform the clinical skills as they were away from the institution for a longer time (20.6% SA and 62.4% A). They also reported that the inability to incorporate the theory into the practice hindered their learning (15.7% SA, 68.6% A) and develop the clinical decision-making skills (15.2% SA, 68.6% A).

Due to the abrupt shift from the face-to-face to the e-learning education, there were concerns about the availability of technology in terms of the devices used for attending the remote instructions, internet access, and skills in the use of technology. Based on the socioeconomic status, the students used the devices for the remote learning, and hence the quality of the device they used would vary. The researchers have found that when the students expected to use the electronic devices for the remote instructions, classwork, online reading, and assignments, the problem with the technology caused stress and affected the academic performance of the students ( Gonzales *et al.*, 2020). The data from the present study revealed that 70% of the participants agreed that the software used for the remote learning was user-friendly and 69% of them agreed that the teachers’ technical skills helped them a lot to have uninterrupted classes. However, contradicting that 53% of them perceived that unfamiliarity with the use of technology made the remote learning difficult. Along with the technical issues, there were the major challenges such as network issues (52% A and 34.5% SA), electricity issues and bad weather during the remote learning. One of the significant factors that favoured the remote learning was the availability of teaching-learning material and the recordings of the classes on the same platform (72%).

The perceptions of remote learning conducted among 804 medical students from Poland reported various advantages and disadvantages. The majority (73%) of students enjoyed the remote learning. Learning at the students’ own pace (64%), ability to stay at home (69%), continuous access to online material (69%), and the comfortable environment were reported as the advantages of the remote learning whereas, lack of patient interaction (70%) and technology-related problems (54%) were the disadvantages that the medical students faced. The remote learning was considered less effective in honing the skills and the social competencies (p<0.001) than the face-to-face learning whereas there was no difference in knowledge gain (p=0.46). However, the students were less active during the remote teaching than the traditional classes ( Bączek *et al.*, 2020). In the present study, 71.5% of them expressed that the remote instruction timings were convenient than the classroom learning. Mukhtar *et al.* (n.d.), reported that the remote learning encouraged student-centred learning. They could learn asynchronously at any time of the day and become self-directed learners. In the present study, most of them (65%) agreed that their self-directed learning skills had improved.

As per the responses from the student nurses who participated in the present study, 61.5% agreed that the remote classes provided enough opportunity to clarify the doubts with the teacher and they even agreed (70.7%) that the teachers did motivate them being away from the institution. The majority of the (56.2%) participants agreed that the teachers were able to give attention to the individual students as in the physical classroom. More than half of them (53%) perceived that being away from the institution for a longer duration and attending the classes remotely was a stressful situation for them. They perceive that being away from the regular classes affected the overall development (57.6%). Even though they perceived it as a stressful situation, the majority (61.9%) agreed that the instructions given during the remote classes were clear as in the physical classroom. The most of the participants (66%) agreed that the remote teaching mode gave an adequate opportunity for the instructor feedback for the students as in the physical classes.

Accessibility is a major concern in the remote teaching learning in most countries. The students from the remote areas faced connectivity problems. The English language education study programme in Indonesia identified that the availability and the sustainability of internet connection, accessibility of teaching media, and compatibility of the device used to access the media were the major obstacles for the remote learning. Hence, to increase the student participation in the remote learning there should be more friendly platforms ( Agung & Surtikanti, 2020). A study conducted by Baloran (2020) reported that due to poor internet connectivity the college students refused to have a blended online learning approach during the COVID-19 pandemic. The majority students (61.78%) preferred learning inside the classroom than remotely. This finding was also supported by Hassan Ja’ashan (2015). Subedi *et al.* (2020) reported that 63.2% of students were affected because of electricity and 63.6% internet problem, only 64.4% of the students had the internet access for their remote classes. Khalil *et al.* (2020) reported that poor internet connectivity and utility of the online tools were the challenges of the remote learning. In the present study, 86.2% of students reported that the connectivity issue (34.5% SA and 51.7% A) was a major concern during the classes, especially in the rural area.

The COVID-19 pandemic had a psychological and emotional impact and affected the students’ mental health ( Lee, 2020). The students of the institutions of higher education, including nursing, experienced moderate to severe stress and anxiety in the virtual classrooms during the COVID-19 pandemic ( AlAteeq *et al.*, 2020; Gallego-Gómez *et al.*, 2020; Sheroun *et al.*, 2020). The findings of the present study also support this, where 52.9% agreed and 19% strongly agreed that they were stressed being away from the institution for a long time and attending the classes remotely. Providing a high-quality remote teaching learning using a stable educational framework and encouraging the students may help in lowering their anxiety ( Savitsky *et al.*, 2020). The perceptions of the students from a university of higher education from India reported that the remote learning helped them continue their learning and complete the syllabus. As the students were not accustomed to learning with cell phones or computers, it led to a lack of interest and attention during the remote classes. The students were unable to attend a few classes when the data limit exceeded for the day ( Mishra *et al.*, 2020). In the present study, the majority of students agreed that they were able to accomplish the learning objectives (72.8% A, 8.6% DA).

The students found that if the recorded classes were available during the remote classes, it helped in better understanding the information whereas if the concepts were difficult it was harder to follow ( Khalil *et al.*, 2020). Deepika (2020) reported that the quality and the timely interaction between the student and the teacher, availability of technical support, a structured module for the remote classes, and facilities for conducting practical classes were essential for the student and the teacher satisfaction with the remote instructions. Most of the students (66.2%) agreed that they had an opportunity for the instructor feedback during the remote teaching.

The present study findings revealed that among the student nurses who participated, 62% agreed and 29% of them strongly agreed that the remote learning was safer amidst the COVID pandemic and 53% of them agreed that the remote classes were interesting, and they enjoyed learning. The majority of them (65.0%) agreed that the new teaching environment improved self-directed learning skills. They perceived it as they needed to be more accountable (74.2%) and responsible (72.6%) to achieve the learning outcomes during the pandemic pedagogy. The half of the participants (51.0%) perceived no difference between the remote and the face-to-face classroom learning, as both helped them to understand the course concepts in the same manner. Nearly half of them (49%) agreed that a virtual demonstration of nursing procedures helped them to develop clinical skills as in the regular teaching. The students agreed that being away from the clinical setting for a long time affected the clinical skills (59%) and felt they were not confident in performing the procedures that they missed to practice (62.4%).

A study conducted to explore the challenges to remote medical education during the COVID-19 pandemic reported that pandemic related stress, issues related to the online experience, communication, and time management were the challenges faced ( Rajab *et al.*, 2020). The challenges faced by the teachers may influence the students’ remote learning. A qualitative study conducted among the teachers in India reported that the home environment with family interruptions, external distractors, lack of basic facilities, lack of support from the institution on training and support for the use of technology, and lack of technical knowledge as the barriers faced by the teachers during the remote teaching ( Joshi *et al.*, 2020). The present study also reported several challenges such as poor internet connectivity, inability to understand the concepts, lack of peer support, an opportunity for group study, and lack of opportunity for the clinical practice.

This study has several strengths. Firstly, the study tried to explore how nursing students perceived the remote learning during the COVID-19 pandemic. This gives information to the institutions of nursing education to understand the views of the students. Secondly, the challenges identified allow the universities to overcome these factors and deliver quality nursing education. Thirdly, since the study is conducted in a multicentre with a large number, generalizability of the findings to all the nursing institutions in India is possible. Nonetheless, this study also has a few limitations. Firstly, since it was a cross-sectional study the detailed information on the challenges could not be explored. Secondly, since the study has not been focused on assessing the learning, it would help improving the assessment methods.

The implications of the study are as follows. Firstly, the emergency remote teaching-learning was the first experience for the students as well as the teachers and this study has explored the student’s perception towards this pandemic pedagogy. Secondly, various challenges that hindered the learning of students need to be deliberated by the institutions and the government so that we prepare well for such pandemics in the future.

## Conclusions

The COVID-19 pandemic posed significant concerns among the students of higher education institutions. The results from the present study conclude that the students prefer the remote learning as well as face-to-face classes. Though remote learning only could be adopted during the pandemic, to achieve adequate clinical skills the students need to get clinical experience with adequate simulated learning and direct patient care. The nursing institutions must adopt blended learning to achieve competencies needed keeping in mind the control of the spreading COVID-19 infection. In addition, the challenges of the pandemic COVID-19 lead to prospects for the development of nursing education and provide quality education by adopting the technology.

## Data availability

### Underlying data

The undelying data set is available at: Assariparambil, Anil Raj (2022): Final_Pandemic_Study.xlsx. figshare. Dataset. https://doi.org/10.6084/m9.figshare.19170041.v1
