# Pandemic Pedagogy: Perception of Nursing students’: A cross-sectional study

**DOI:** 10.12688/f1000research.109789.3

**Published:** 2023-09-26

**Authors:** Prima Jenevive Jyothi D'Souza, Anil Raj Assariparambil, G Muthamilselvi, Veena M Joseph, Linu Sara George

**Affiliations:** 1 Assistant Professor, Department of Fundamentals of Nursing, Manipal College of Nursing Manipal, Manipal Academy of Higher Education, Udupi district, Karnataka, 576104, India; 2 Assistant Professor, Department of Medical Surgical Nursing, Manipal College of Nursing Manipal, Manipal Academy of Higher Education, Udupi District, Karnataka, 576104, India; 3 Professor and principal, Vinayaka Mission’s College of Nursing, Vinayaka Mission’s Research Foundations,, Puducherry, Tamil Nadu, India; 4 Assistant Professor, College of Nursing, Gulf Medical University, Ajman, United Arab Emirates; 5 Professor & Head, Department of Fundamentals of Nursing, Manipal College of Nursing Manipal, Manipal Academy of Higher Education, Udupi district, Karnataka, 576104, India

**Keywords:** Nursing, Perception, Pandemics, Students, Teaching

## Abstract

Coronavirus disease 2019 pandemic impacted across the globe disrupting all sectors including the higher education universities. Nursing institutions faced various challenges due to the pandemic restrictions, of which the abrupt shift of implementing the curriculum to online mode posed a major challenge to both the teachers and the students. To assess nursing students’ perception of pandemic pedagogy and the challenges faced in online teaching-learning, this cross-sectional survey was conducted among 982 undergraduate nursing students from three Deemed to be University nursing institutions of Southern India. Institutional Ethics Committee approval (IEC 444/2020), permission from the heads of the institutions and study participant’s consent was obtained. Data was collected using an online survey questionnaire which had three domains, including student-related (19 items), teacher-related(5 items), and physical learning environment-related factors (11 items). The reliability was established using Cronbach’s Alpha (0.86). Explored the favouring, hindering factors and challenges faced during the emergency remote teaching with open-ended items. The overall mean score of perceptions on pandemic pedagogy was 89.03±10.03. Sixty-three percent of students had a total perception score above 87 which indicates that they preferred online learning during the pandemic whereas 45% preferred classroom learning. There was a significant difference in the total perception scores and the years of study( F (3, 978) = 4.96, p = 0.002). The factors favouring online learning were, an opportunity to view the recorded classes even after the live classes’ (n=165), and ‘more time to spend for learning activities’ (n=152). Factors that hindered the learning or the challenges faced were poor network connectivity (n=451), and lack of opportunity for group study (n=326). Students favoured online learning during the pandemic; however, there were several challenges. The educational institutions need to prepare themselves to overcome this and focus on a blended learning curriculum.

## What is already known about the topic?

• The COVID-19 pandemic has disrupted educational activities worldwide.
• Higher education institutions, including nursing, have quickly moved to remote teaching-learning for uninterrupted education.
• There is a gap in the knowledge of emergency remote teaching for the nursing curriculum during the COVID-19 pandemic.

## What does this paper add?

• This study describes how nursing students perceived remote learning and the challenges faced during the COVID-19 pandemic.
• The findings of the study contribute to nursing education, emphasizing the need for remote teaching-learning for uninterrupted learning and preventing the spread of infection by staying safe at home.
• The incorporation of blended learning post-pandemic helps nurse educators and policymakers to take appropriate steps in escalating nursing education in the country to greater heights.

## Introduction

The outbreak of COVID-19 hampered educational activities worldwide. Educational institutions in most countries had to engage in remote teaching learning from face-to-face teaching during and after the lockdown. When the COVID-19 crisis would end in uncertainty and colleges started functioning as before, it led to continuing e-learning in many parts of the world indefinitely until the pandemic came under control.

What we experienced during COVID-19 was unprecedented. Nursing institutions faced a unique challenge as they had a significant role in bringing up the next generation of care providers ( Dewart *et al.*, 2020). The COVID-19 pandemic has pushed us towards remote learning from traditional classroom learning ( Hodges *et al.*, 2020). The sudden shift from face-to-face classes to remote learning due to the pandemic posed challenges for teachers and students due to the technology and its accessibility. Nursing education focuses on the development of knowledge and clinical competency for providing quality care to patients. During the course of study, theory and practicums go hand-in-hand for students, bridging the gap between theory and practice. Students acquire their clinical skills by practicing in both laboratories and hospitals. Clinical experiences are vital for developing technical expertise and enhancing procedural skills in nursing. Nursing education emphasizes the significance of both technical skills and critical thinking in nursing practice and clinical judgement. Therefore, while clinical experiences contribute to skill development, critical thinking remains an integral aspect of nursing education and practice for effective problem-solving and decision-making. As the government declared the closure of educational institutions due to the pandemic, students had to move to their homes without knowing about their return to the colleges. Both students and teachers assumed that they would return at the earliest, but the pandemic kept them in their homes for more than six months.

All the nursing schools in India quickly moved on to emergency remote teaching from face-to-face classes. The institutions adopted synchronous and asynchronous technology based on convenience and availability. The synchronous technology included live interactions between teachers and students during online classes, whereas the asynchronous technology had a time gap between the instructor and its recipient ( Finkelstein, 2006). Implementing emergency remote teaching helped students continue learning outside educational institutions, keeping students and teachers safe in their homes. During this phase, students might have had varied experiences and faced various challenges due to changes in the learning environment. In India before the pandemic, most of the teaching-learning activities occurred either in formal classrooms or laboratories under the teacher’s constant mentoring, whereas in the mode of emergency remote teaching, the students had to take responsibility for learning themselves.

In 2021, Dvořáková *et al.* conducted a study on how university students perceived remote learning during the first wave of the COVID pandemic. The study included 68 undergraduates and 31 postgraduates students who were surveyed online through MS forms. Out of 99 participants, the average satisfaction score for emergency remote teaching was 4.07 out of 5. Eighty percent of students revealed that during remote learning students missed the opportunities for classroom discussions and interaction with classmates and teachers. Regarding their stress level during remote learning, 48% expressed that they were partly stressed, and 19% expressed being more stressed than in regular face-to-face teaching.

Emergency remote teaching or pandemic pedagogy in nursing and other professions has not been much extensively explored. The objective of the study was to assess the perceptions of the undergraduate students of nursing on pandemic pedagogy and identify the challenges they faced in remote learning.

## Methods

### Design and sample

This cross-sectional survey was conducted among undergraduate nursing students studying in the deemed to be university nursing institutions of southern India, under the University Grants Commission of India.

### Setting and data collection

Investigators obtained approval from the Institutional Ethics Committee (IEC 444/2020) for conducting the study and permission from the heads of the institutions. The study was registered under the clinical trial registry of India (CTRI/2020/08/027047).

All the deemed to be universities nursing institutions (13 institutions) of southern India were approached and included only those three nursing institutions permitted to conduct the study. Students undergoing a four-year undergraduate nursing degree programme were included in the study. The students were sent home from the institutions under lockdown, and all three nursing institutions moved on to emergency remote teaching. Since the institutions did not have remote teaching before the pandemic, it was a sudden change for both teachers and students. Initially, teachers themselves explored various platforms and later they were oriented to remote teaching platforms such as Microsoft teams, Impartus and Edmodo by the institutions. The nursing institutions offered approximately three to five hours of remote instruction during the pandemic.

An online survey with a written consent form was sent to all the participants via Google Form to their contact details submitted to the institution. The students consented to participate in the study after reading the participant information sheet provided in the form.

### Sample size and sampling technique

**Figure 1.**
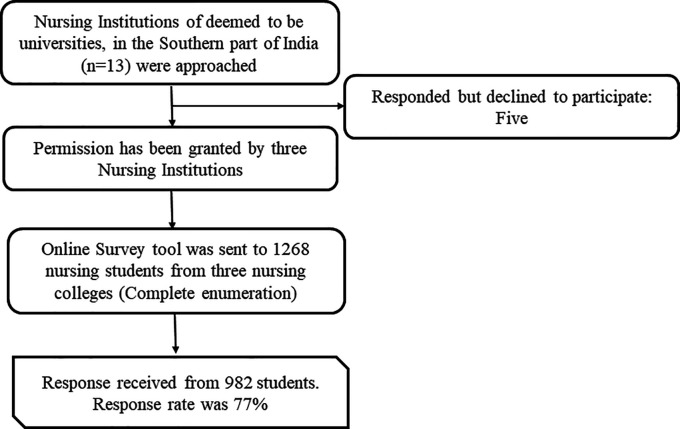
A schematic flow chart representing sample selection.

A complete enumeration of all the nursing institutions deemed to be universities in southern India was the sampling technique considered in the study. However, out of 13 nursing institutions of the Deemed Universities in the selected geographical area, three nursing colleges agreed to participate in the study. The online survey questionnaire was sent to all the students of the three colleges, and they were requested to participate in the study if they were willing to Figure 1 depicts the schematic flow chart representation of the sample selection.

### Variables and measures

#### Demographic

A demographic questionnaire was used to collect the information on age, year of study, area of residence, mode of access to remote teaching learning, and the device used.

#### Perception of pandemic pedagogy

Students perception of pandemic pedagogy were assessed using a four-point Likert scale. It consisted of 35 items with four open-ended questions. The questionnaire had three domains, including student-related (19 items), teacher-related (5 items), and physical learning environment-related factors (11 items). The response options for each item were: strongly agree (SA), agree (A), disagree (D), and strongly disagree (SD) with scores of 4, 3, 2, and 1, respectively. The total score ranged from 35 to 140, with a cut-off of > 87 as an indicator of favouring remote learning. Scores less than 86 indicated that the students favoured face-to-face classroom learning during the pandemic. The open-ended questions explored the favouring factors and the challenges students faced during emergency remote teaching.

The investigators developed this survey questionnaire after reviewing the available literature and taking opinions from the students. It was validated by five experts from the fields of nursing (three) and medical education (two). The scale content validity index (S-CVI) was computed based on the experts’ agreement, and the questionnaire was valid (S-CVI: 0.82). As per the suggestions from the experts a few modifications were made in the final tool. The reliability was established by administering the scale to 20 post-basic BSc. nursing students, and Cronbach’s alpha computed was 0.86.

### Statistical analysis

Data cleaning was carried out using Microsoft Excel for irrelevant observations, inaccuracies, and unwanted outliers before analysis in SPSS. There were no missing data as all mandatory items were completed in the Google Form. The data analysis of the study was carried out using SPSS version 16 (SPSS, RRID:SCR_002865). The demographic variables and the perceptions of the students on remote learning were expressed in frequencies and percentages. The mean and the standard deviation of the perception score based on the year of study are described. To find the difference in the perception score between the years of study, ANOVA was used and post hoc comparisons were performed using the Hochberg’s GT2 test. Independent sample t-test were performed to find the difference between the areas of residence and the perception score. The factors facilitating pandemic pedagogy and the challenges faced were described in frequency.

## Results

### Sample characteristics

The characteristics of the nursing students who participated in the pandemic pedagogy perception survey are summarized in Table 1. The mean age of the student nurses was 19.8 years ( *SD*=1.4). The common platforms used by the institutions for the remote pedagogy were Zoom (34.3%), MS Teams (34.3%), Webex (8.2%), Google Meet (7.2%), Edmodo (7.1%), Impartus (5.8%), and Google Classroom (2.7%).

**Table 1.** A description of sample characteristics (N=982).

Sample characteristics	Frequency (f)	Percentage (%)
*Year of study*		
First	127	12.9
Second	236	24.0
Third	248	25.3
Fourth	***371***	***37.8***
*Area of residence*		
Rural	***530***	***54.0***
Urban	432	46.0
*Type of device used for remote learning*		
Mobile phone	***866***	***88.2***
Computer	20	2.0
Tablet	6	0.6
Both Mobile phone and Computer	90	9.2
*Network coverage at the place of stay*		
Good	206	21.0
Average	***651***	***66.3***
Poor	125	12.7
*The orientation is given by the institution on the use of the remote teaching platform*		
Yes	***888***	***90.4***
No	94	9.6

### Nursing students’ perception of pandemic pedagogy

The overall mean score of the perceptions of students on pandemic pedagogy was 89.03±10.03. The year-wise mean scores are given in Table 3. Sixty-three percent of students had a total perception score above 87, indicating that they preferred remote learning during the pandemic, whereas 45% preferred face-to-face learning in the classroom. The response for the individual items on the perception scale is summarized in Table 2.

**Table 2.** Description of nursing students’ perceptions of pandemic pedagogy (N=982).

Sl. No.	Item	Perception			
		Strongly agree (SA)	Agree (A)	Disagree (D)	Strongly disagree (SD)
		f (%)	f (%)	f (%)	f (%)
	***Students related factors***				
1.	Remote classes helped me to accomplish the learning objectives.	84 (8.6)	715(72.8)	129 (13.1)	54 (5.5)
2.	I was able to concentrate better during the remote classes compared to the face-to-face classes.	53 (5.4)	354 (36.0)	425 (43.3)	150 (15.3)
3.	The new teaching environment improved my self-directed learning skills.	71 (7.2)	638 (65.0)	227 (23.1)	46 (4.7)
4.	The remote classes were more interactive than the classroom learning.	48 (4.9)	320 (32.6)	465 (47.4)	149 (15.2)
5.	The remote class timings were convenient than the classroom learning.	2 (0.2)	702 (71.5)	213 (21.7)	65 (6.6)
6.	I was able to communicate with my classmates well during this pandemic time like that in the classroom.	85 (8.7)	428 (43.6)	362 (36.9)	107 (10.9)
7.	I never got a sense of isolation by attending the remote classes being away from the regular classroom.	83 (8.5)	564 (57.4)	262 (26.7)	73 (7.4)
8.	Being away from the regular classroom, I needed to have more accountability to achieve the learning outcomes.	103 (10.5)	729 (74.2)	126 (12.8)	24 (2.4)
9.	Being away from the regular classroom, I needed to take more responsibility to achieve the learning outcomes.	158 (16.1)	713 (72.6)	86 (8.8)	25 (2.5)
10.	The remote learning helped me to understand the course concepts as good as the regular classroom.	74 (7.5)	501 (51.0)	319 (32.5)	88 (9.0)
11.	Virtual demonstration of nursing procedures helped me to develop my clinical skills as in the regular teaching.	67 (6.8)	484 (49.3)	328 (33.4)	103 (10.5)
12.	Being away from the clinical setting I felt I am not confident of performing the procedures that I missed to practice.	202 (20.6)	613 (62.4)	123 (12.5)	44 (4.5)
13.	My clinical skills have been affected by being away from the clinical setting for a longer time.	259 (26.4)	577 (58.8)	114 (11.6)	32 (3.3)
14.	Being away from the clinical setting, I could not incorporate the theory into practice and it hindered my learning.	154 (15.7)	665 (67.7)	133 (13.5)	30 (3.1)
15.	I could not develop the clinical decision-making skills being away from the clinical setting.	154 (15.7)	674 (68.6)	129 (13.1)	25 (2.5)
16.	Being away from the institution for a long duration and attending classes remotely has made me stressed.	187 (19.0)	519 (52.9)	222 (22.6)	54 (5.5)
17.	Engaged in the remote learning, I am worried about the depth of my knowledge of the course content.	149 (15.2)	634 (64.6)	169 (17.2)	30 (3.1)
18.	Being away from the regular classes has affected my overall development.	161 (16.4)	566 (57.6)	212 (21.6)	43 (4.4)
19.	The regular classroom learning gave opportunities for the extracurricular activities.	214 (21.8)	619 (63.0)	107 (10.9)	42 (4.3)
	***Teacher related factors***				
20.	The remote classes gave me enough opportunity to clarify doubts with the teacher.	121 (12.3)	604 (61.5)	205 (20.9)	52 (5.3)
21.	Teachers motivated me during the remote classes although I was away from the institution.	176 (17.9)	694 (70.7)	85 (8.7)	27 (2.7)
22.	During the remote classes, the teacher was able to give attention to the individual students as in the physical classroom.	80 (8.1)	552 (56.2)	272 (27.7)	78 (7.9)
23.	The instructions given during the remote classes were clear as in the physical classroom.	85 (8.7)	608 (61.9)	232 (23.6)	57 (5.8)
24.	The teacher kept the learners active throughout the class.	101 (10.3)	701 (71.4)	138 (14.1)	42 (4.3)
	***Physical learning environment-related factors***				
25.	The remote learning was safer amid the COVID pandemic.	288 (29.3)	608 (61.9)	56 (5.7)	30 (3.1)
26.	The remote classes kept me engaged so much that I did not have time to think unnecessarily about the COVID-19.	153 (15.6)	627 (63.8)	157 (16.0)	45 (4.6)
27.	The mode of remote teaching gave adequate opportunities for the instructor feedback as in the physical classes.	88 (9.0)	650 (66.2)	194 (19.8)	50 (5.1)
28.	The home atmosphere was not suitable for the teaching and learning process.	164 (16.7)	475 (48.4)	243 (24.7)	100 (10.2)
29.	The software used for the remote learning was user friendly.	134 (13.6)	690 (70.3)	115 (11.7)	43 (4.4)
30.	The remote learning allowed sharing the teaching-learning material on the same platform.	139 (14.2)	711 (72.4)	100 (10.2)	32 (3.3)
31.	Network/internet issues interfered with my remote learning.	339 (34.5)	508 (51.7)	96 (9.8)	49 (4.0)
32.	Unfamiliarity with the use of technology made the remote learning difficult.	107 (10.9)	519 (52.9)	274 (27.9)	82 (8.4)
33.	The use of the learner engagement applications (Kahoot, Mentimeter, etc.) kept me active during the remote class.	125 (12.7)	581 (59.2)	233 (22.7)	43 (4.4)
34.	The technical skills of the teachers helped to have an uninterrupted classes.	108 (11.0)	678 (69.0)	158 (16.1)	38 (3.9)
35.	The remote classes were interesting, and I enjoyed learning.	102 (10.4)	524 (53.4)	261 (26.6)	95 (9.7)

### A comparison of nursing students’ perception of pandemic pedagogy with years of study

The three domains included in the perception tool were student-related, teacher-related, and physical learning environment-related factors. This section of the article discusses the comparison of domain wise analysis with the students as per their years of study. The comparison table depicted in Table 3 reveals no changes between the mean values of the domain scores across the different years of study. To compare nursing students’ perception of pandemic pedagogy with years of study, ANOVA was computed, and it revealed that there was a significant difference ( *M*=89.03, *SD*=10.03, F (3, 978)=4.96, p=0.002) in the total perception scores and the years of study. The post hoc comparisons using Hochberg’s GT2 test indicated that the mean perception score of the first-year students ( *M*=86.48, *SD*=10.95) was significantly different than that of the second-year students ( *M*=90.97, *SD*=9.83). However, the third ( *M*=89.43, *SD*=9.11) and fourth-year ( *M*=88.65, *SD*=10.26) students’ perception scores did not significantly differ from those of the first and second-year students.

**Table 3.** A comparison of nursing students’ perceptions of pandemic pedagogy mean scores by years of study (N=982).

Year of study	Student-related factors	Teacher-related factors	Physical learning environment-related factors	Overall	Mean Square	df	f	p
	(Min 19-Max 76)	(Min 5-Max 20)	(Min 11-Max 44)	(Min 35-Max 140)				
	Mean±SD	Mean±SD	Mean±SD	Mean±SD				
1 ^st^ Year *(n=127)*	43.89±5.87	13.59±2.56	29.31±4.04	86.80±10.12	493.75	3,978	4.96	0.002 ^*^
2 ^nd^ Year *(n=236)*	46.0±5.26	14.51±2.64	30.24±3.43	90.95±9.27				
3 ^rd^ Year *(n=248)*	45.51±5.87	14.02±2.37	29.37±3.92	88.91±9.80				
4 ^th^ Year ( *n=371)*	45.28±6.25	14.06±2.53	29.30±3.50	88.65±10.39				
Overall ( *N=982)*	45.38±5.91	14.10±2.54	29.54±3.68	89.03±10.03				

^*^ Significant at the level of p<0.05.

### Comparison of nursing students’ perception of pandemic pedagogy with the area of residence

A comparison of the perceptions of the nursing students on pandemic pedagogy with the area of residence has been described in Table 4. To make the comparison an independent sample ‘t’ test was used. The findings reveal that there is no significant difference in the perception scores of the students residing in rural ( *M*=88.68, *SD=*9.86) and urban ( *M*=89.43, *SD=*10.23) areas; t (1.16), p=0.243.

**Table 4.** A comparison of perceptions of nursing students on pandemic pedagogy with the area of residence (N=982).

Area of residence	n	Mean±SD	df	t	p
Rural	530	88.68±9.86	980	1.16	0.243
Urban	452	89.43±10.23			

### Factors favouring pandemic pedagogy

The participants were asked to express their views on the factors that favoured remote learning during the COVID-19 pandemic. The responses are summarized descriptively, and the major aspects revealed were: ‘an opportunity to view the recorded classes even after the live classes’ (n=165), ‘more time to spend for learning activities’ (n=152), ‘ample opportunity to clarify doubts like physical classroom’ (n=139) and ‘able to concentrate well as the teacher is directly interacting’ (n=110). Other opinions were as follows; “ *flexible and convenient class timings motivated self-directed learning, study materials were made available immediately after each lecture helped in completing studies on time, in the current situation of a pandemic being along with parents were relaxing and stress-free and which helped in better learning, training sessions arranged by the college helped a lot in handling the remote teaching-learning platform efficiently.”*

### Challenges faced during pandemic pedagogy

A descriptive summarization of the responses regarding the factors that hindered learning or the challenges faced in remote teaching-learning during the COVID-19 pandemic is discussed in this section. The majority of nursing students responded that internet issues, either due to poor network coverage, bad weather or monsoons, were a major concern as they could not understand the topics discussed (n=451). The participants also quoted a few other challenges: ‘could not have group study and discussion which was practiced in the college campus’ (n=326), ‘Clinical skills cannot be taught well through the remote teaching platforms’(n=315), ‘attending the classes through the mobile phone was difficult as often getting tired (headache, eye issues) due to the constant use’ (n=262), ‘less motivation to learn being away from the college’ (n=205), and ‘not able to concentrate being at home’ (n=135). Some of them also mentioned the *unavailability of library facilities and fewer opportunities to maintain interpersonal relationships with teachers and friends* as the challenges faced during this pandemic time.

### Opinion on future directives for classes during the postpandemic period

Participants were asked to express their opinion on having a few hours of remote instruction along with face-to-face classes during the academic year post pandemic. The descriptive summary of the open-ended questions reveals that the future directions on the remote instructions were inconclusive. Most of them preferred to be at safe places during the pandemic and continue with remote learning. Under the favouring factors and the challenges faced during remote instruction, there is a mixture of opinions about the future direction of remote learning. As the curriculum demands hands-on clinical experience through direct patient care, they opined that it was a major missing. A few of them even suggested that the theory hours in the future could be a mixture of both online and offline classes, whereas a few felt that there were a lot of negative impacts of remote learning since it had pros and cons. To list a few physical ill effects, no opportunity for cocurricular activities, less opportunity to be together and interact with friends and teachers. Most of them expressed that although there were pros and cons for the remote instruction, the greatest challenges were network issues, the impact of weather on electricity, and data connectivity.

## Discussion

This study explores undergraduate nursing students’ perceptions of remote learning during the COVID-19 pandemic. The sudden shift to remote learning presented challenges for teachers and students unprepared for this transition. Remote teaching-only classes may affect student performance, especially for those who are already struggling academically. Face-to-face instruction enables the academic performance of students more than remote teaching learning ( Xu & Jaggars, 2014). The present study reveals that 81.4% of the participants (72.8% A and 8.6% SA) agreed that the remote instructions helped to accomplish the learning objectives. However, 64.6% of the students were concerned about their lack of in-depth understanding of the subject. The majority of students (43.3% D, 15.3% SD) disagreed that remote instruction helped them concentrate better than face-to-face teaching. Additionally, they found remote instruction less interactive than classroom learning (47.4% D, 15.2% SD). Half of the participants (51.0%) perceived no difference between remote and face-to-face classroom learning, as both helped them to understand the course concepts in the same manner.

In the present study, there was a mixed response from the participants on the aspect of opportunity to communicate with classmates well during this pandemic time similar to what happened in the classroom (43.6% A, 36.9% D). Most of them (57.4 % A, 8.5% SA) agreed that they never got a sense of isolation while attending the remote classes away from the regular classroom and 63% agreed that the opportunities for extracurricular activities were greater in the regular classroom learning. Bali and Liu (2018) explored higher education students’ perceptions and indicated that social presence, social interaction, and satisfaction were higher in face-to-face learning than in remote learning. However, a few students preferred remote learning, as the technology led them to be innovative. The student engagement remains a challenge in the remote learning.

Engaging students and enhancing student-learning may not be as effective in remote learning as in classroom learning. The students may not be able to discuss and have doubts that arise during the remote classes cleared ( Alturise, 2020). In the present study, the students perceived that they were kept active throughout the class (71.4% A, 10.3% SA) with the use of learner engagement applications (59.2% A, 12.7% SA).

Remote learning would hinder the acquisition of clinical skills. A study conducted among dental students and staff reported that the closure of teaching clinics affected students’ clinical competence ( Loch *et al.*, 2020). In the present study, most students felt that they were not confident in performing the clinical skills as they were away from the institution for a longer time (20.6% SA and 62.4% A). They also reported that the inability to incorporate the theory into the practice hindered their learning (15.7% SA, 68.6% A) and development of the clinical decision-making skills (15.2% SA, 68.6% A). Nearly half of them (49%) agreed that a virtual demonstration of nursing procedures helped them to develop clinical skills as in regular teaching. The students agreed that being away from the clinical setting for a long time affected their clinical skills (59%) and felt they were not confident in performing the procedures that they missed to practice (62.4%).

Due to the abrupt shift from face-to-face to remote teaching-learning education, there were concerns about the availability of technology in terms of the devices used for attending remote instruction, internet access, and skills in the use of technology. The type of devices used by students for remote learning varied based on their socioeconomic status. The researchers found that when students expected to use electronic devices for remote instruction, classwork, online reading, and assignments, the problem with the technology caused stress and affected the academic performance of the students ( Gonzales *et al.*, 2020). The data from the present study revealed that 70% of the participants agreed that the software used for remote learning was user-friendly and 69% of them agreed that the teachers’ technical skills helped them to have uninterrupted classes. However, 53% of them perceived that unfamiliarity with the use of technology made remote learning difficult. Along with technical issues, there were major challenges such as network issues (52% A and 34.5% SA), electricity issues and bad weather during remote learning. One of the significant factors that favoured remote learning was the availability of teaching-learning material and the recordings of the classes on the same platform (72%).

A study conducted among 804 medical students from Poland reported various advantages and disadvantages of remote learning. The majority (73%) of students enjoyed remote learning. Learning at the students’ own pace (64%), ability to stay at home (69%), continuous access to online material (69%), and a comfortable environment were reported as the advantages of remote learning whereas, a lack of patient interaction (70%) and technology-related problems (54%) were the disadvantages that the medical students faced. Remote learning was considered less effective in honing skills and social competencies (p<0.001) than face-to-face learning whereas there was no difference in knowledge gain (p=0.46). However, the students were less active during remote teaching than during traditional classes ( Bączek *et al.*, 2020). In the present study, 71.5% of them expressed that remote instruction timings were more convenient than classroom learning. Mukhtar *et al.* (n.d.), reported that remote learning encouraged student-centered learning. They could learn asynchronously at any time of the day and become self-directed learners. In the present study, most of them (65%) agreed that their self-directed learning skills had improved. They also perceived that, they needed to be more accountable (74.2%) and responsible (72.6%) to achieve learning outcomes during the pandemic pedagogy.

As per the responses from the student nurses who participated in the present study, 61.5% agreed that the remote classes provided enough opportunity to clarify their doubts with the teacher and they even agreed (70.7%) that the teachers did motivate them to be away from the institution. The majority of the (56.2%) participants agreed that the teachers were able to give attention to the individual students as in the physical classroom. More than half of them (53%) perceived that being away from the institution for a longer duration and attending classes remotely was a stressful situation for them. They perceived that being away from regular classes affected their overall development (57.6%). Even though they perceived it as a stressful situation, the majority (61.9%) agreed that the instructions given during the remote classes were as clear as those in the physical classroom. Most of the participants (66%) agreed that the remote teaching mode provided an adequate opportunity for instructor feedback for the students as in the physical classes.

Remote teaching and learning in many countries face challenges in regard to accessibility. Students living in remote areas often encounter problems with connectivity. The English language education program in Indonesia found that the availability and sustainability of internet connections, accessibility of teaching materials, and compatibility of devices were major obstacles to remote learning. To increase student participation in remote learning, it is important to have more user-friendly platforms available ( Agung & Surtikanti, 2020). According to a study by Baloran (2020), due to poor internet connectivity, college students refused to have a blended online learning approach during the COVID-19 pandemic. The majority of students (61.78%) preferred learning inside the classroom rather than remotely. This finding was also supported by Hassan Ja’ashan (2015). Subedi *et al.* (2020) reported that 63.2% of students were affected because of electricity and 63.6% because of internet problems, only 64.4% of the students had internet access for their remote classes. Khalil *et al.* (2020) reported that poor internet connectivity and the utility of online tools were the challenges of remote learning. In the present study, 86.2% of students reported that connectivity issues (34.5% SA and 51.7% A) were a major concern during classes, especially in rural areas. A study conducted to explore the challenges to remote medical education during the COVID-19 pandemic reported that pandemic related stress, and issues related to the online experience, communication, and time management were the challenges faced ( Rajab *et al.*, 2020). The challenges faced by teachers may influence students’ remote learning. A qualitative study conducted among teachers in India reported that the home environment with family interruptions, external distractors, lack of basic facilities, lack of support from the institution on training and support for the use of technology, and lack of technical knowledge were the barriers faced by teachers during remote teaching ( Joshi *et al.*, 2020). The present study also reported several challenges such as poor internet connectivity, inability to understand the concepts, lack of peer support, lack of opportunity for group study, and lack of opportunity for clinical practice.

The COVID-19 pandemic had a psychological and emotional impact and affected students’ mental health ( Lee, 2020). Students in institutions of higher education, including nursing, experienced moderate to severe stress and anxiety in virtual classrooms during the COVID-19 pandemic ( AlAteeq *et al.*, 2020; Gallego-Gómez *et al.*, 2020; Sheroun *et al.*, 2020). The findings of the present study also support this, where 52.9% agreed and 19% strongly agreed that they were stressed by being away from the institution for a long time and attending classes remotely. Providing high-quality remote teaching learning using a stable educational framework and encouraging students may help lower their anxiety ( Savitsky *et al.*, 2020). The perceptions of the students from a university of higher education from India reported that remote learning helped them continue their learning and complete the syllabus. As the students were not accustomed to learning with cell phones or computers, it led to a lack of interest and attention during the remote classes. The students were unable to attend a few classes when the data limit was exceeded for the day ( Mishra *et al.*, 2020).

The students discovered that having access to recorded classes during remote learning improved their understanding, while difficult concepts made it harder to follow ( Khalil *et al.*, 2020). According to Deepika (2020), it is crucial for both students and teachers to have quality and timely interactions, technical support, structured remote modules, and practical class facilities for satisfactory remote instruction. Most of the students (66.2%) agreed that they had an opportunity for instructor feedback during remote teaching.

The present study findings revealed that among the student nurses who participated, 62% agreed and 29% of them strongly agreed that remote learning was safer amidst the COVID pandemic and 53% of them agreed that remote classes were interesting, and that they enjoyed learning.

This study has several strengths. First, the study tried to explore how nursing students perceived remote learning during the COVID-19 pandemic. This gives information to nursing education institutions to understand the views of the students. Second, the challenges identified allow universities to overcome these factors and deliver quality nursing education. Third, since the study was conducted in multicentre with a large number, generalizability of the findings to all nursing institutions in India is possible. Nonetheless, this study also has a few limitations. First, since it was a cross-sectional study detailed information on the challenges could not be explored. Second, since the study has not been focused on assessing learning, it would not help improve the assessment methods.

The implications of the study are as follows. First, the emergency remote teaching-learning was the first experience for the students as well as the teachers and this study explored the student’s perceptions of this pandemic pedagogy. Second, various challenges that hindered the learning of students need to be deliberated by institutions and the government so that we can prepare well for such pandemics in the future.

## Conclusions

This study on nursing students’ perceptions of pandemic pedagogy offers valuable insights into the challenges and experiences faced during remote teaching and learning during the COVID-19 pandemic. The results from the present study conclude that an abrupt shift to remote learning during the COVID-19 pandemic led to disruption in teaching and learning activities. Students perceived various challenges during remote learning, especially in gaining clinical skills. Exploring students’ perceptions highlights the need for flexible and innovative approaches, integrating digital technologies, and emphasizing student engagement. As the healthcare landscape rapidly evolves, nursing education must continue to evolve as well, incorporating lessons from the pandemic era. The experiences shared in the study emphasize the importance of fostering resilience, adaptability, and technological proficiency among nursing students. These findings have broader implications for nursing education beyond the pandemic, guiding the development of future-ready curricula that prepare nurses to navigate complex healthcare scenarios with confidence.

## Data availability

### Underlying data

The undelying dataset is available at: Assariparambil, Anil Raj (2022): Final_Pandemic_Study.xlsx. figshare. Dataset. https://doi.org/10.6084/m9.figshare.19170041.v2 ( Assariparambil *et al.*, 2022).
