# Supplementary material for: Pandemic Pedagogy: Perception of Nursing students’: A cross-sectional study
Source: F1000Res. 2023 Sep 26;11:398. Originally published 2022 Apr 6. [Version 3] doi: 10.12688/f1000research.109789.3 (PMC10579851; doi:10.12688/f1000research.109789.3)
Supplement: Peer review history

# Reviewer report: Reviewer response for version 3

**DOI:** 10.5256/f1000research.155566.r209583

**Published:** 2023-10-16

**Authors:** Carlos Laranjeira

**Affiliations:** 1 School of Health Sciences, Polytechnic Institute of Leiria, Leiria, Portugal

Thank you for attending to the concerns raised by the reviewers across multiple revisions. The quality of the manuscript has been greatly improved as a result of the changes that you have made.

 I have some additional comments which focus primarily on improving the clarity of the paper:

- The paper does not cite an actual and appropriate range of literature sources. Relevant literature should be presented more deeply in order to support the research problem.
- Please provide the specific questions/hypotheses under study, it would be useful for the readers.

Method

- In your methods section, say that you used the STROBE cross-sectional reporting guidelines.
- When the data were collected? Data collection period?
- A rationale is needed for the sampling procedure used.
- Some subjects refused to participate. Response rate?
- The process of analysis should be made as transparent as possible. What strategies were used to avoid duplications or fraud in the online survey? Did you analyze any potential non-response bias? And early vs late bias? Did you check if data can suffer from common method bias?

Discussion

- A stronger discussion of implications for future research and potential intervention work is needed.
- Theoretical and methodological limitations should be emphasized more deeply.

Is the work clearly and accurately presented and does it cite the current literature?

No

If applicable, is the statistical analysis and its interpretation appropriate?

Yes

Are all the source data underlying the results available to ensure full reproducibility?

No

Is the study design appropriate and is the work technically sound?

Yes

Are the conclusions drawn adequately supported by the results?

No

Are sufficient details of methods and analysis provided to allow replication by others?

No

Reviewer Expertise:

NA

I confirm that I have read this submission and believe that I have an appropriate level of expertise to confirm that it is of an acceptable scientific standard, however I have significant reservations, as outlined above.

# Reviewer report: Reviewer response for version 2

**DOI:** 10.5256/f1000research.136425.r191731

**Published:** 2023-09-07

**Authors:** Carlos Laranjeira

**Affiliations:** 1 School of Health Sciences, Polytechnic Institute of Leiria, Leiria, Portugal

I appreciate the opportunity to review this manuscript and hope my comments assist in the revision process. The material is interesting and the topic is timely and relevant. The method seems to have been followed faithfully and the authors were well-positioned to conduct the analysis. Despite these positives, in my view, the paper needs more work before it could be published and I have made some specific suggestions below.

- The paper does not cite an actual and appropriate range of literature sources. Relevant literature should be presented more deeply in order to support the research problem. Further, there is no clear distinction between manuscript sections in terms of the content they report. First, I suggest dividing the section "INTRODUCTION" into three components, respectively introduction (explain the general argument of the paper, without going into specific details) background (situate the study concepts within the context of extant knowledge, discuss the international relevance of the concepts) and purpose, creating greater clarity in the analysis of the reader. What is the study's biggest contribution? The contribution should be clearly stated in the introduction.
- As a reader, I was a bit confused with regard to the purpose addressed in this study. Please provide the specific questions/hypotheses under study, it would be useful for the readers.

Method

- In your methods section, say that you used the STROBE cross-sectional reporting guidelines.
- When the data were collected? Data collection period?
- Provide a clear description of the method used to select the participants for the study (e.g. convenience or consecutive). A rationale is needed for the sampling procedure used.
- Some subjects refused to participate. Response rate?
- Were the sample sizes sufficiently powered to detect effects? How did the researchers decide on sample size?
- Was there compensation for participating in the study?
- Please cite the literature that supported the development of the data collection instrument. The validation of instrument needs more detail.
- The process of analysis should be made as transparent as possible. What strategies were used to avoid duplications or fraud in the online survey? Did you analyze any potential non-response bias? And early vs late bias? Did you check if data can suffer from common method bias?

Results

- How authors handled missing data?

Discussion

- Some of the contributions that are highlighted here could be flagged in the introduction for a more consistent narrative throughout the paper. Discussion can be improved if questions/hypotheses will be outlined in the introduction, then answered suitably in this section, and the results interpreted appropriately. I believe there should be better integration of the results with the existing literature.
- A stronger discussion of implications for future research and potential intervention work is needed.
- Theoretical and methodological limitations should be emphasized more deeply.

CHECKLIST FOR STYLE

- The manuscript will serve a broad audience of students, researchers, and practitioners, however, the manuscript needs to be carefully and attentively proofread, because some sentences are awkwardly constructed, punctuation is deficient, and therefore reading is occasionally difficult to follow. That leads me to believe that it needs careful editing by a native English speaker.

Is the work clearly and accurately presented and does it cite the current literature?

No

If applicable, is the statistical analysis and its interpretation appropriate?

Yes

Are all the source data underlying the results available to ensure full reproducibility?

No

Is the study design appropriate and is the work technically sound?

Yes

Are the conclusions drawn adequately supported by the results?

No

Are sufficient details of methods and analysis provided to allow replication by others?

No

Reviewer Expertise:

Mental health, vulnerable groups, end-of-life care, quantitative reserach, qualitative research

I confirm that I have read this submission and believe that I have an appropriate level of expertise to state that I do not consider it to be of an acceptable scientific standard, for reasons outlined above.

# Reviewer report: Reviewer response for version 2

**DOI:** 10.5256/f1000research.136425.r182787

**Published:** 2023-08-09

**Authors:** Isaac Amankwaa

**Affiliations:** 1 Waikato Institute of Technology, Hamilton, New Zealand

Dear Authors,

 Thank you for your manuscript exploring the perceptions of students on remote learning during the COVID-19 pandemic. This study is both timely and relevant, given the shift in educational methodologies worldwide. However, there are several areas that would benefit from revisions to enhance clarity, precision, and readability. Please find the summarised feedback below:

**Abstract **

- While mentioning the ethical approval for your study is essential, it isn't necessary to specify the approval number (IEC 444/2020) in the abstract. A general statement of approval from an Institutional Ethics Committee should suffice. Also, please ensure to use the standardized term "COVID-19" instead of "Corona Virus-2019".

**Introduction**

- **Accuracy of Statements:** The sentence, " *Getting adequate clinical experiences would improve the skills in performing a nursing procedure, rather than enhancing critical thinking and clinical decision-making abilities*", may need to be revised. It's generally accepted that clinical experience can foster both technical skills and cognitive skills such as critical thinking and clinical decision-making. You might want to reconsider this statement or provide robust supporting evidence for it.
- **Use of Citations:** Please provide references to support certain claims made in your introduction. For instance, when you state, " *Before the pandemic, most of the teaching-learning activities occurred either in formal classrooms or laboratories under teacher’s constant mentoring, whereas in the mode of emergency remote teaching, the students had to take the responsibility for learning themselves,*" it would be helpful to cite literature supporting these observations. If these observations are based on anecdotal evidence or specific to the Indian context, please clarify this.
- **Contextual Understanding:** The sentence, " *COVID-19 pandemic had pushed us towards e-learning from traditional classroom learning,*" may require more context or elaboration. While it's accurate that the pandemic hastened the transition towards e-learning, the trend towards online education had already been gaining momentum before the pandemic, particularly in countries like the United States. It would be beneficial to modify this statement to reflect the broader context.
- **Substantiated Claims:** You mention, " *The abrupt interruption of the well-planned academic activities in the face-to-face classes and the shift to remote teaching and learning was challenging for both teachers and students due to the technology and its accessibility.*" This sentence seems to make an assumption about the state of academic activities before the pandemic. Were all academic activities indeed well-planned before COVID-19? It would be beneficial to back this claim with evidence or rephrase it to avoid making potentially unfounded assumptions.

**Methods**

- **Missing Data**: please indicate if there were any missing data and how such cases were handled in the analysis. It is important to disclose this information as it affects the interpretation of your findings. Please consider providing information on how missing data were managed in the study.
- **Response Rate:** For such a large sample with survey delivered online, it would be useful to report the response rate. This is important to assess the representativeness of your sample and understand the generalizability of your findings. Please provide this information.
- **Data Preparation:** How was data prepared for analysis? Did you clean the data before the analysis? If so, what processes did you follow? Providing this information enhances the transparency of your study and allows for the replication of your study by other researchers.
- **Instrument Validation:** You mentioned that nursing and medical education experts validated the survey questionnaire. However, further details on this process would be useful. How many experts reviewed the questionnaire? What was their feedback? Was any revision made based on their feedback?
- **Sample Selection Description:** Your description of the sample selection process seems a bit unclear. When describing that only three out of 13 institutions accepted to participate, was this due to the institutions not providing permission, or was it due to other reasons? Clarification on this point would help readers understand the context of your study.

**Results**

 The data is well presented and analyzed. 

- You have included the p-values for the tests you conducted, but there are places where the description of significance is not clear. For instance, for the independent sample ‘t’ test between rural and urban students, you report a p-value of 0.0243. While this is generally considered significant, you reported no significant difference. Please clarify or correct this.

**Discussion**

- Your discussion comprehensively covers the students' experiences and compares them with previous studies, providing depth to the topic. However, every reference used should contribute directly to your findings or show how your work expands upon previous research. Please present your findings in a more simplified manner to aid reader understanding. Avoid repetition of similar points across the paper to prevent redundancy. Finally, ensure that your conclusion explicitly states your main findings and their implications for nursing education in the context of COVID-19.

**General flow and clarity of manuscript**

 There are several areas in your manuscript that could benefit from further refinement to enhance clarity, precision, and readability. Here are my compiled comments:

- **Grammar and Language Usage:** The manuscript occasionally exhibits grammatical errors, inappropriate word choices, and awkward sentence constructions. These issues disrupt the smooth reading of the content and may confuse readers. For instance, "The most students" should be corrected to "Most students". I recommend comprehensive proofreading of the manuscript to identify and correct such errors.
- **Clarity:** There are instances where the ideas conveyed are unclear or difficult to understand due to complex sentence structures. Simplifying sentences and using active voice more often could help with this. For example, " *The perceptions of remote learning conducted among 804 medical students from Poland reported various advantages and disadvantages*" might be better expressed as "A study conducted among 804 medical students from Poland reported various advantages and disadvantages of remote learning".
- Be consistent in capitalising the phrase of " *Three Deemed to be University nursing institutions of Southern India*". There are areas where this is in small case and in other areas, the are upper cases.

Is the work clearly and accurately presented and does it cite the current literature?

Partly

If applicable, is the statistical analysis and its interpretation appropriate?

Partly

Are all the source data underlying the results available to ensure full reproducibility?

Yes

Is the study design appropriate and is the work technically sound?

Partly

Are the conclusions drawn adequately supported by the results?

Partly

Are sufficient details of methods and analysis provided to allow replication by others?

Partly

Reviewer Expertise:

Nursing Education

I confirm that I have read this submission and believe that I have an appropriate level of expertise to confirm that it is of an acceptable scientific standard, however I have significant reservations, as outlined above.

# Reviewer report: Reviewer response for version 2

**DOI:** 10.5256/f1000research.136425.r145576

**Published:** 2022-08-11

**Authors:** Karen E. Alexander

**Affiliations:** 1 Department of Clinical and Applied Sciences-Nursing, University of Houston–Clear Lake, Houston, TX, USA

The revisions have provided the clarity needed. As such it is ready for indexing. It is a very timely and interesting article as well.

Is the work clearly and accurately presented and does it cite the current literature?

Partly

If applicable, is the statistical analysis and its interpretation appropriate?

I cannot comment. A qualified statistician is required.

Are all the source data underlying the results available to ensure full reproducibility?

Yes

Is the study design appropriate and is the work technically sound?

Yes

Are the conclusions drawn adequately supported by the results?

Yes

Are sufficient details of methods and analysis provided to allow replication by others?

Partly

Reviewer Expertise:

My research focus is on vulnerable populations (fathers of preterm infants, veterans, adolescent food insecurity) and Nursing Education curriculum and delivery.

I confirm that I have read this submission and believe that I have an appropriate level of expertise to confirm that it is of an acceptable scientific standard.

# Reviewer report: Reviewer response for version 1

**DOI:** 10.5256/f1000research.121337.r135191

**Published:** 2022-06-13

**Authors:** Karen E. Alexander

**Affiliations:** 1 Department of Clinical and Applied Sciences-Nursing, University of Houston–Clear Lake, Houston, TX, USA

**Abstract: **Regarding online learning, what was the students preferred online learning (synchronous/asynchronous)? This should be specified to provide clarity.

**Sample**: The sample characteristics would flow better under demographics instead of results.

**R** **emote versus Online instruction:**  Some parts of the article (i.e. the introduction) refer to “online” and other sections speak to “remote” teaching. Moreover, the authors speak to students sent home to learn online, it seems from the information provided, that they were learning remotely.  Since there is a difference between remote versus online instruction this needs to be explicated.

*Remote instruction* is most often a solution to facilitating a course curriculum in times of face-to-face class interruptions (e.g., a scheduled absence, an inclement weather day, or an emergency). In the case of this article, one would assume due to the pandemic. This form of instruction is typically designed in a responsive manner and often delivered through the instructor’s preferred technology at the time of delivery to best meet course outcomes under the given circumstance. *On the other hand, Online instruction* is the facilitation that occurs in a course that has been developed with the intention of fully online delivery. Instructional experiences are designed in a planned manner, over weeks and months, most often with the support of an instructional designer and a media services team. The learning experiences and instructional objects in an online course are typically fully-developed before the start of a semester. In other words, remote teaching occurs when the instructor, transitions the delivery of an on-campus course to online. An online course, however, has been purposely designed for online teaching using online learning design principles. Because the 2 terms are used interchangeably in this article it is difficult the understand what was actually the mode of delivery for the students.

**Introduction:** The reference “An e-newsletter ‘education week’ reported the opinion of the high school students from France concerning online learning.” Does not seem to reflect or compare to deemed university nursing students. Suggest a reference to students in another similar university setting as a freshman.

**Technology Platform:** Regarding the platform used *“Initially, teachers themselves explored various platforms and later they were oriented to online teaching platforms by the institutions too.”* This leaves the question. What was the platform used?

**Results/Discussion:** The following information needed to be explicated earlier - “ *The institutions adopted synchronous and asynchronous technology based on convenience and availability.*

*The synchronous technology included live interactions between teacher and students during online classes, whereas the asynchronous had a time gap between the instructor and its recipient*.” Again, this needed to be explicated earlier in learner engagement application descriptions to understand the modality.

**References**

 References were applicable and up-to-date to support the background and results.

 Summary, Overall good article. Interesting and timely.

Is the work clearly and accurately presented and does it cite the current literature?

Partly

If applicable, is the statistical analysis and its interpretation appropriate?

I cannot comment. A qualified statistician is required.

Are all the source data underlying the results available to ensure full reproducibility?

Yes

Is the study design appropriate and is the work technically sound?

Yes

Are the conclusions drawn adequately supported by the results?

Yes

Are sufficient details of methods and analysis provided to allow replication by others?

Partly

Reviewer Expertise:

Nursing education, vulnerable populations, and health disparities.

I confirm that I have read this submission and believe that I have an appropriate level of expertise to confirm that it is of an acceptable scientific standard, however I have significant reservations, as outlined above.

# Response

**Published:** 2022-07-20

**Authors:** Anil Raj Assariparambil

**Affiliations:** Manipal College of Nursing, Manipal Academy of Higher Education, India

We appreciate the reviewer’s effort in systematically cross-checking the manuscript and for your valuable suggestions.

1. **Abstract:** Students favored synchronized online learning during the pandemic; however, there were several challenges. (We have modified it as per the reviewer’s comment)
2. **Sample:** *"The sample characteristics would flow better under demographics instead of results." *
  Respected reviewer, we are not very clear about this comment. However, is that before the results section, do we need to add a separate heading as sample demographics? We usually describe the sample demographics under the results section. Hence, we would like to retain the same format in the manuscript.
3. **Remote versus Online instruction:** *"Some parts of the article (i.e. the introduction) refer to “online” and other sections speak to “remote” teaching."* 
  Thank you for notifying us with the detailed description. It was really an eye-opener! We have changed it to remote instruction instead of online.
4. **Introduction**: Citation and reference were updated as per suggestion
5. **Technology Platform:  **Modified the sentence mentioning the platforms
6. **Results/Discussion:** The following information needed to be explicated earlier - “ *The institutions adopted synchronous and asynchronous technology based on convenience and availability.* 
  We have incorporated this statement into the introduction

Once again thank you so much for you generous inputs to modify the manuscript. 

 Thanking you 

 With Regards
